# Real-time prediction of stomach motions based upon gastric contraction and breathing models

**DOI:** 10.1088/1361-6560/ac9660

**Published:** 2022-12-16

**Authors:** Yuhang Zhang, Yue Cao, Rojano Kashani, Theodore S. Lawrence, James M. Balter

**Affiliations:** 1 Department of Radiation Oncology, University of Michigan; 2 Department of Biomedical Engineering, University of Michigan; 3 Department of Radiology, University of Michigan; 4 Department of Radiation Oncology, University Hospitals Seidman Cancer Center

**Keywords:** gastric contraction motion, Magnetic Resonance Imaging, motion prediction

## Abstract

**Objective:**

Precision radiation therapy requires managing motions of organs at risk that occur during treatment. While methods have been developed for real-time respiratory motion tracking, non-breathing intra-fractional variations (including gastric contractile motion) have seen little attention to date. The purpose of this study is to develop a cyclic gastric contractile motion prediction model to support real-time management during radiotherapy.

**Approach:**

The observed short-term reproducibility of gastric contractile motion permitted development of a prediction model that 1) extracts gastric contraction motion phases from few minutes of golden angle stack of stars scanning (at patient positioning), 2) estimate gastric phase of real-time sampled data acquired during treatment delivery to these reconstructed phases and 3) predicting future gastric phase by linear extrapolation using estimation results from step 2 to account for processing and system latency times. Model was evaluated on three parameters including training time window for step 1, number of spokes for real-time sampling data in step 2 and future prediction time.

**Main Results:**

The model was tested on a population of 20-minute data samples from 25 scans from 15 patients. The mean prediction error with 10 spokes and 2 minutes training was 0.3±0.1 mm (0.1 – 0.7 mm) with 5.1 s future time, slowly rising to 0.6±0.2 mm (0.2 – 1.1 mm) for 6.8 s future time and then increasing rapidly for longer forward predictions, for an average 3.6±0.5 mm (2.8 – 4.7 mm) HD95 of gastric motion. Results showed that reducing of train time window (5 to 2 min) doesn’t influence the prediction performance, while using 5 spokes increased prediction errors.

**Significance:**

The proposed gastric motion prediction model has sufficiently accurate prediction performance to allow for sub-millimeter accuracy while allowing sufficient time for data processing and machine interaction and shows the potential for clinical implementation to support stomach motion tracking during radiotherapy.

## 1. Introduction

Precision treatment of intra-abdominal tumors and concomitant sparing of surrounding normal tissues is complicated due to various motions that influence abdominal configuration[1–4]. While breathing is the most significant and relevant motion in the abdomen and has received a lot of attention[5], additional motions, for example those arising from stomach contractions, may influence both tumor as well as organ at risk (OAR) positions and configurations dynamically during treatments. Recent investigations of adaptive therapy treatments have demonstrated the potential for changes in abdominal configuration to impact radiation dose delivery to tumors and surrounding normal tissues [6, 7], but the assessment of such changes has been limited to samples acquired either on successive treatment days or otherwise image volumes acquires at two or three time points during a treatment delivery. A current lack of methods to assess the impact of continuous non-breathing motions on abdominal OARs leads to either ignoring these effects completely (and thus risking both tumor underdose as well as normal tissue overdose) or otherwise applying generic margins to OARs for safety to limit their maximum dose (with possible impacts of underdose or overdose depending on the relationship of these margins to actual motions). Methods of monitoring contractile motion may prove beneficial for MR-guided precision therapies, e.g., when delivering treatment to targets proximal to the stomach. In our previous paper, we demonstrated that representing breathing and gastric contraction as independent motion sources yielded accurate motion models [8]. This investigation also demonstrated both the magnitudes as well as short-term reproducibility of contractile motion in our patient population, who were for clinical purposes instructed to not eat solid foods for at least 2 hours prior to scanning. We have recently determined that contractile motion has significant influence on the position of tumors and other OARs within a few cm of the stomach [9]. Treatment monitoring and motion management techniques that capitalize on the independent nature of contractile motion in the body not only improve our ability to deliver more precise radiation treatment but may also provide significant insight into developing robust treatment planning strategies.

Integrated MRI and linear accelerator systems (“MR-Linacs”) provide a potentially effective infrastructure for managing intra-fractional motions through quite flexible continuous sampling capability during treatment delivery. Such systems are routinely used to monitor breathing motion and turn the beam off (gate) transiently for motions outside of selected boundaries. To date, however, the primary focus of these monitoring methods has been applied either generically to any observed changes (typically using two-dimensional images), or to specifically focus on breathing-related effects. Meanwhile, little attention has been paid to non-breathing driven-motion effects such as gastric motions during MR-guided treatment delivery[10, 11]. Several studies have shown the ability of online adaptive abdominal radiotherapy to account for inter-fraction geometric uncertainties in treatment sites such as liver [7], pancreas [6, 7, 10, 12, 13], and prostate [11], providing image guided gastric motion monitoring to such systems may improve the ability to improve treatment outcomes.

One limitation of MR-Linacs is the latency between image acquisitions and gating or adjustment of the treatment machines. Latency arises due to time needed to decide on appropriate actions as well as time for the linear accelerator control systems to implement any such decisions. The latter time has recently been reported to be around 250 to 500ms [14]. Any motion monitoring and decision strategy would need to include time needed for prediction in addition to system reaction latency to support accurate motion management. Motion prediction frameworks have been implemented to address these combined latency issues when monitoring breathing motion by predicting near-future motion to guide gating or machine adjustments [15, 16]. Breathing motion prediction methods have been extensively studied, and several methods have been explored to address this problem, including linearized regression model, nonlinear estimation model, Kalman filtering and artificial neural networks [17–21].

Our group has recently investigated methods to predict slow configuration changes in the abdomen in parallel with breathing motions [22]. In this paper, we propose a gastric motion prediction methodology, to allow for real-time management of gastric contraction during radiation treatments to achieve better precision of dose delivery and organ-at-risk (OAR) avoidance, nominally with an uncertainty of 1 mm or less. The prediction framework takes advantage of the observed short-term high regularity of the gastric contractile cycle.

## 2. Gastric Contraction Motion Characterization

### 2.1 Data Collection and Preparation

Under an Institutional Review Board-approved protocol, 15 patients underwent a total of 25 sessions of Dynamic Contrast-Enhanced (DCE-) MR Imaging, in which a 20-minute time series was acquired using a golden-angle stack-of-stars gradient-echo sequence on a 3T scanner (Skyra, Siemens Healthineers, Erlangen, Germany), yielding 7,000 radial stacks of spokes in k-space from each scan. The field of view covered the liver, stomach and a large portion of the intestines. The imaging parameters included echo time values of 1.14 to 1.21 ms, repetition times of 2.71–4.51 ms, flip angles of 10°to 14°, in-plane voxel sizes from 2–2.45 mm and slice thicknesses of 3–4 mm. The size of the imaging matrix was 192 × 192 and the number of slices was 64. Each radial stack of spokes will be referred to as a ‘spoke’ for simplicity in the following sections.

### 2.2 Gastric Motion Extraction

The gastric contraction motion phase signals for each spoke were extracted using a recently published method [9]. In brief, following breathing motion correction of spokes [23], a time series of high temporal and low spatial resolution images was reconstructed using a view-sharing filter of spokes, with $\sigma_{\text{min}}$ and $\sigma_{max}$ equal to 1 and 5 spokes. $\sigma_{min}$ and $\sigma_{max}$ indicate minimum and maximum temporal widths of the view-sharing filter. A region of interest encompassing the stomach, contoured by an expert on a vendor-reconstructed reference image, was propagated to these time-series images. A Rician-distribution filter (equation 1), designed to emphasize the approximate frequency window of gastric contraction in humans, was applied to the intensity matrix of the stomach from this time series of images

(1) $$f((x|s,\sigma )=I_{0}(\frac{xs}{\sigma^{2}})\frac{x}{\sigma^{2}}e^{-(\frac{x^{2}+s^{2}}{2\sigma^{2}})}$$

**Figure 1.**
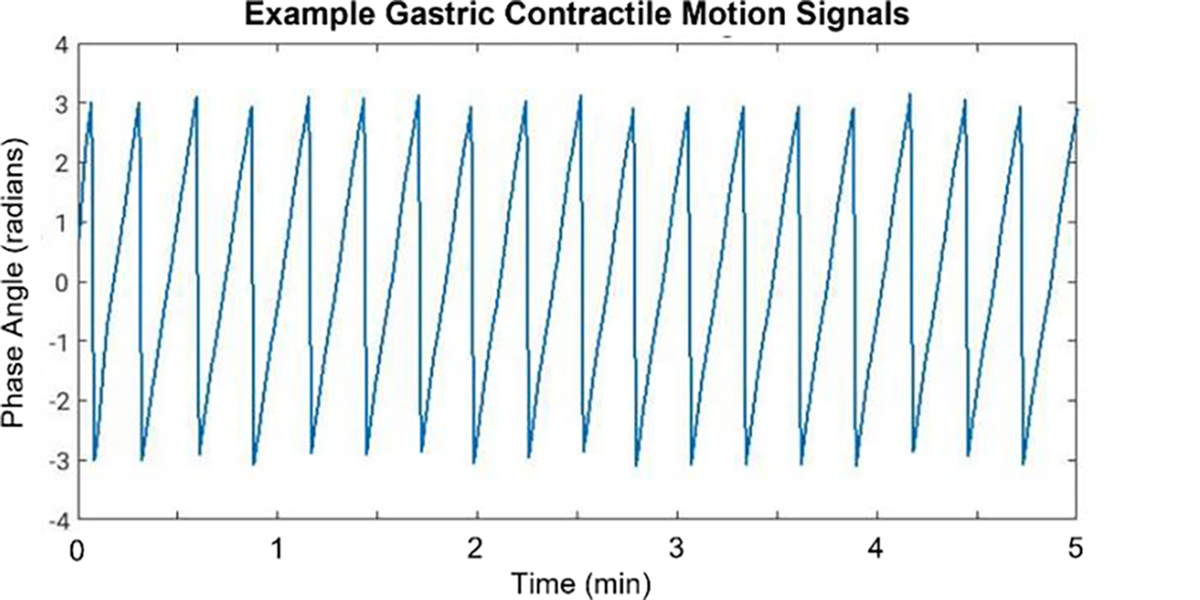
Example of extracted gastric contraction signal over 5 minutes of temporal sampling.

Here, we used the non-centrality parameter s=0.05Hz, and scale parameter σ=0.01Hz.x represent the intensity values, and $I_{0}$ is the zero-order modified Bessel function of the first kind. Following filtering, Principal Component Analysis (PCA) was applied. The phase angle ([−π,π]) between the first principal component and its derivative with respect to itself was extracted and used to define the gastric motion phase. Breathing-frozen spokes were then sorted according to gastric contraction phases and then used to reconstruct 10 phase image volumes using a view sharing filter $\left(\sigma_{min}=700,\sigma_{max}=1400\right)$ along the gastric contractile phase angle dimension. Figure 1 shows an example of extracted motion signals from which the center angles for each state are shown in Table 1.

The phase No. 1 was defined as a reference, to which the other 9 image volumes were deformably aligned to yield deformation fields describing abdominal shape changes due to gastric contraction.

To optimize the algorithm for predicting gastric contractile motions, the temporal stability and magnitude/velocity of the measured contractile motion was explored. The distribution of times between successive peak phases of the gastric motion signal (Δt) was calculated for each of the 25 scans. The distribution of magnitudes of motion of each stomach surface voxel between successive gastric motion phases was determined as well to assess the velocity of possible changes as treatment progresses.

### 2.3 Gastric Motion Prediction Model

To construct the gastric motion prediction model, we assume that, in patients following two hours without food intake, stomach contractions exhibit short-term stability over time windows compatible with MR-guided treatment delivery. We further posit that motion is to first order reproducible across several successive gastric contraction cycles within a treatment fraction.

Under these assumptions, future gastric motion phases can be easily inferred if we know the current motion phase in combination with a fully reconstructed contraction motion model using the data collected either a few minutes earlier, or (not discussed in this preliminary investigation) updated as new information is added during treatment delivery.

**Figure 2.**
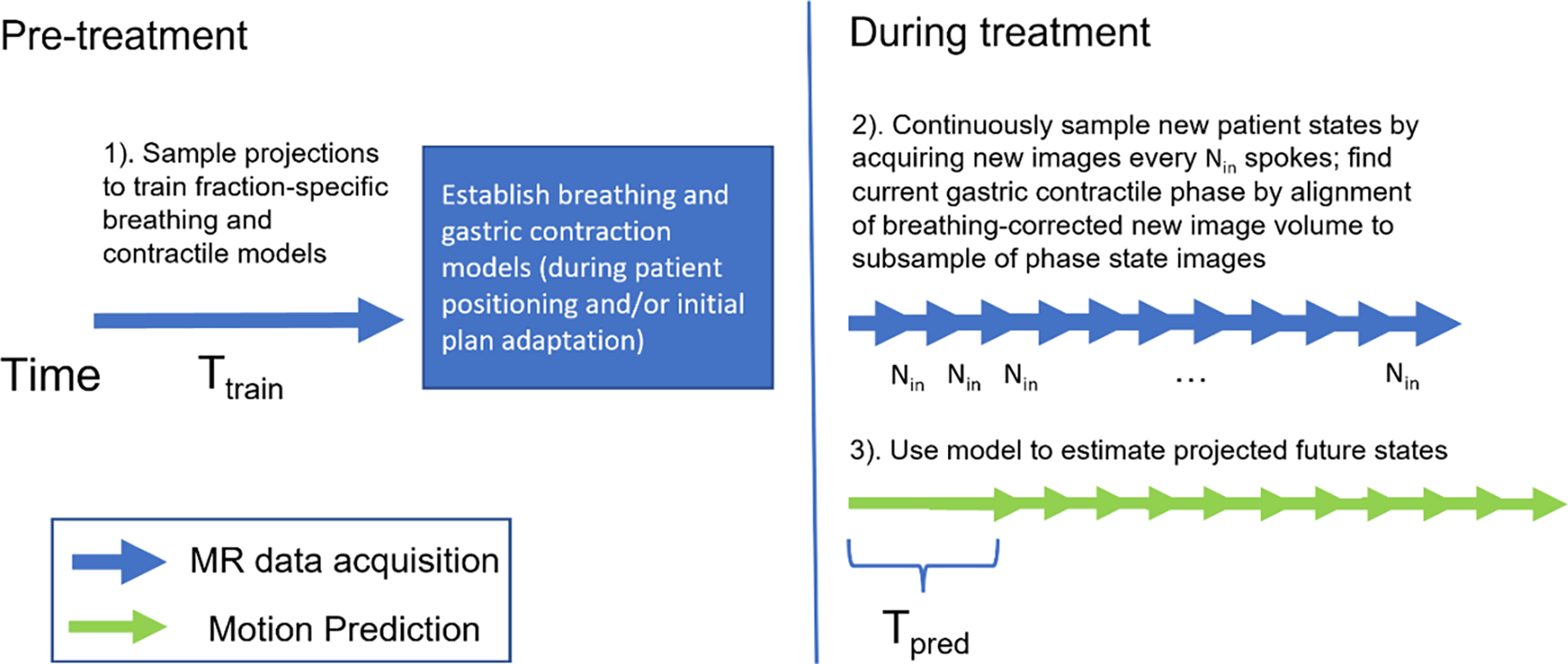
Overview of the prediction model. Three steps are included: 1) gastric contractile phase model generation (training); 2) current phase estimation; 3) Future phase prediction.

A prediction model (Figure 2) was developed that consists of three steps:

#### 1) Gastric Contractile Phase Image Reconstruction (Training)

Gastric contractile motion phase images are reconstructed from spokes sampled (e.g., at the start of a treatment session on a MR-guided linac, possibly for use in patient positioning and/or initial plan adaptation) over a time window of $T_{\text{train}}$ minutes, using the method described in section 2.2. Following breathing motion correction, spokes are sorted according to their gastric motion signals. 10 motion phases are generated by reconstructing along the phase angle with view-sharing, and the number of spokes defined by $T_{\text{train}}$ (e.g., $\sigma_{min}=70,\;\sigma_{max}=140$ for 2 minutes of training data).

#### 2) Current Phase Estimation

Following training, new spokes are continuously sampled. Breathing motion corrected image reconstructions are used to generate reconstructed image volumes every $N_{\text{in}}$ spokes. The breathing signal can be simultaneously used as well for gating purposes at a temporal resolution of ~340 ms as demonstrated in our prior publication [22]. The structural similarity (SSIM) metric is calculated between these images and a subset of contractile motion phase images from step 1) to estimate the current motion phase. The phase image with the maximum SSIM is assigned as the current phase. To improve the computation efficiency, only 1 axial and 1 sagittal plane through the locations with maximum areal intersections with the stomach are used for SSIM computation. Taking advantage of the expected short-term temporal stability of gastric contraction as well as velocity of gastric contractile motions, only a subset of phase states beyond that determined from the prior input image were assumed to likely be possible in the time between observations, and as such a limited search of modeled states is used for estimation of the current contractile phase. This subset was set to the current and next two phases, which were defined based on observations of contractile speed (results described in section 3.1).

#### 3) Future Phase Prediction

Given the stable periodicity of contractile motion, the motion phase at future time $T_{\text{pred}}$ is predicted by extrapolating the current estimated phase from step 2) along the temporal axis of motion from the trained model. To improve prediction accuracy, estimation results from past five states are together used for the extrapolation. Specifically, estimated phase numbers for current plus previous five states $\left[N_{i-5},N_{i-4},\ldots ,N_{i-1},N_{i,}\right]$ are transferred to corresponding center phase angles $\left[\theta_{i-5,}\theta_{i-4},\ldots ,\theta_{i-1},\theta_{i,}\right]$ according to Table 1. Given the future time point $T_{\text{pred}}$, phase angle $\theta_{T}$ is predicted by linear extrapolating the array $\left[\theta_{i-5},\theta_{i-4},\ldots ,\theta_{i-1,}\theta_{i,}\right]$ (projecting back to [−π,π] if $\theta_{T}$ goes beyond the range). Then, gastric contraction phase number $N_{T}$ at time point $T_{\text{pred}}$ is determined from $\theta_{T}$ by the nearest center phase angle in Table 1.

### 2.4 Model Evaluation

To evaluate the model, the 20-minute scans were separated into two parts, training and testing. The training used the spokes from the start of the scans and spanning a time window of $T_{\text{train}}$ and the related samples were used for model building in step 1 of section 2.3. The remaining data was used for real-time input image reconstruction for testing model predictions in step 2.

Ground truth states were defined for the sample data by applying the model to the full 20-minute data set. Phase prediction accuracy and geometric errors were quantified for both the current phase of the patient (estimation), as well as the future projected phase (prediction). Phase prediction accuracy was defined by the frequency with which the model-determined phases matched corresponding ground truth phases. Geometric errors were quantified by the 95% Hausdorff Distance (HD95) of the stomach surface between the estimation/prediction phases and the ground truth states (defined by model-based deformation of the reference phase to the actual spoke label). The stomach contour was delineated on the first phase (chosen as the phase centered at −0.9π) of the reconstructed motion phase images and propagated to all other volumes through a deformation field derived from the deformable image registration with the software package NiftyReg [24].To avoid the influence of extreme outliers, the 95-percentile of the Hausdorff Distance distribution (HD95) was selected to define the error. The mean errors for each scan were computed by averaging the HD95 error through the entire $20-T_{\text{train}}min$ testing data for both estimation and prediction.

For each scan session, the average contraction motion magnitude was determined as the average HD95 between all the stomach contours from each phase to the reference phase. Since gastric motion phases are uniformly distributed in temporal space, this number indicates the average stomach motion during the scan and serves as a useful value for comparison to the prediction error.

The model was optimized and evaluated by modifying 3 different parameters, including:

1. $T_{\text{train}}$, with training times of 2, 3 and 5min, corresponding to 70, 105 and 175 spokes per phase respectively.
2. $N_{\text{in}}$, with 10 spokes (1.7 s sampling time) and 5 spokes (0.8 s sampling time) for sampled input images for contractile state monitoring tested.
3. $T_{\text{pred}}$, with forward prediction times for managing latency of 3.4, 5.1, 6.8, 10.3 and 15.4 seconds.

## 3. Results

### 3.1 Characterization of Temporal Stability and Velocity of Contractile Motion

Gastric contraction patterns demonstrated noticeable variability. Across the 25 scans investigated, the average peak-to-peak Δt was 18.8 seconds with a standard deviation of 2.7 s and an overall range from 13.9 to 25.0 seconds. The inter-scan variation of average contraction cycle speed was also observed to be large for repeat scans of the same patient. For the 6 patients with 2 repeated scans, differences of average peak-to-peak Δt between first and second scans were 0.4, 2.4, 0.7, 0.6, 2.7 and 1.0 seconds; for the 2 patients with 3 scans the average peak differences across scans were 4.9 and 2.9 seconds.

**Figure 3.**
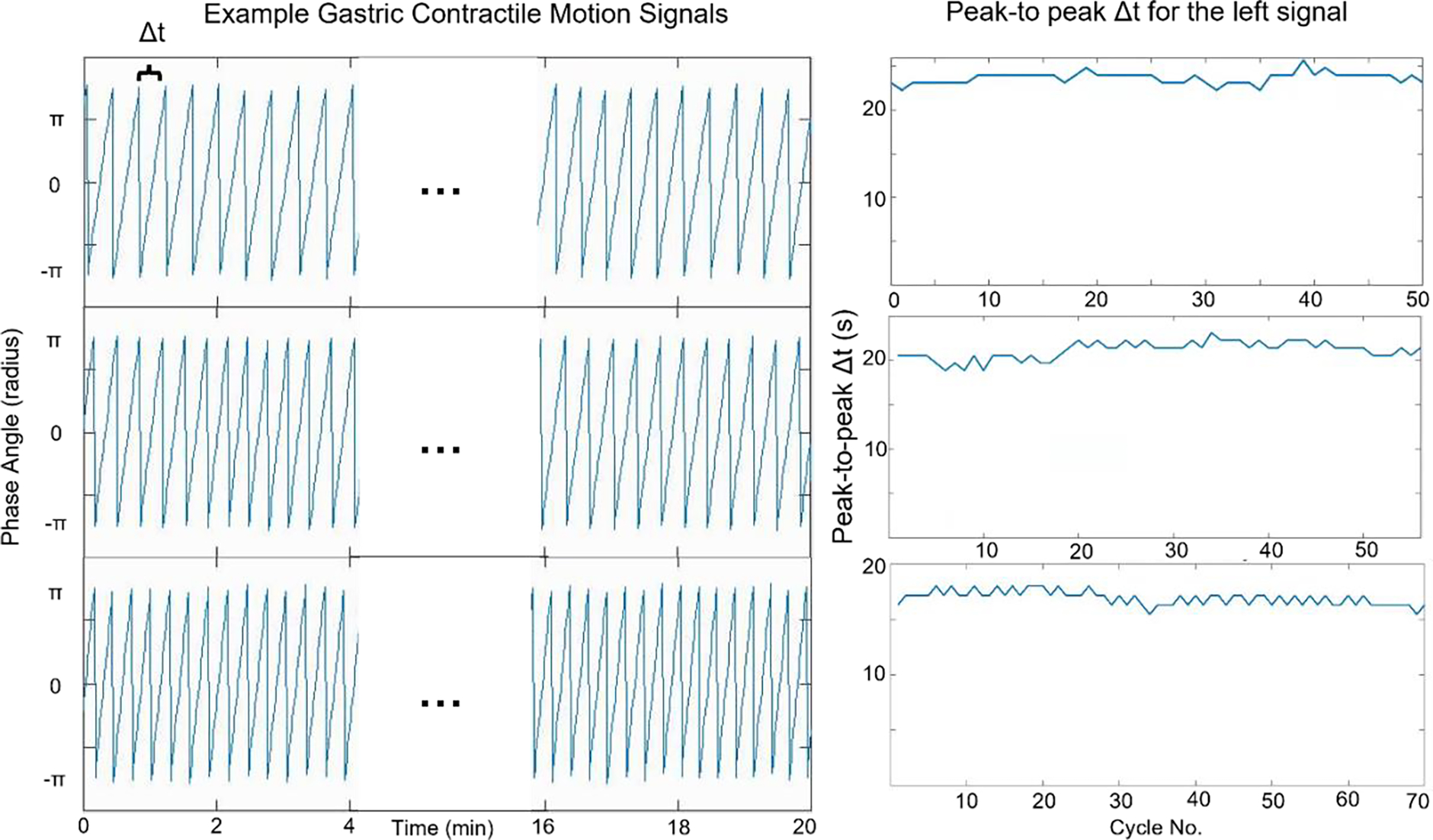
Left: example plots of extracted gastric motion signals from 3 different 20min scans. The x-axis is the time from the start of the scan and y-axis the contractile phase in radians. The average and standard deviation of Δt for the traces shown are 23.1±0.4 s, 21.1±0.6 s and 16.9±0.7 s respectively. Right: computed peak-to-peak Δt values for the examples shown on the left.

Although there were noticeable variations between different scans, the intra-scan peak-to-peak Δt remained very stable for each of the 25 scans. The average standard deviation of intra-scan peak-to-peak Δt across the population was 0.6 seconds, with a range from 0.4 to 0.9 seconds. The maximum range of peak-to-peak Δt variation within any single scan during the whole 20min was found to be 2.4s, which was only observed in one single case. The difference of mean Δt between the first 5-minute (start) and last 5-minute (end) sampling windows of each scan was 0.8±0.3 seconds. Three randomly picked example temporal contraction phase signals were shown in figure 3, along with the peak-to-peak Δt values over the 20-minute window. The high stability of gastric motion within individual scan sessions was illustrated.

**Figure 4.**
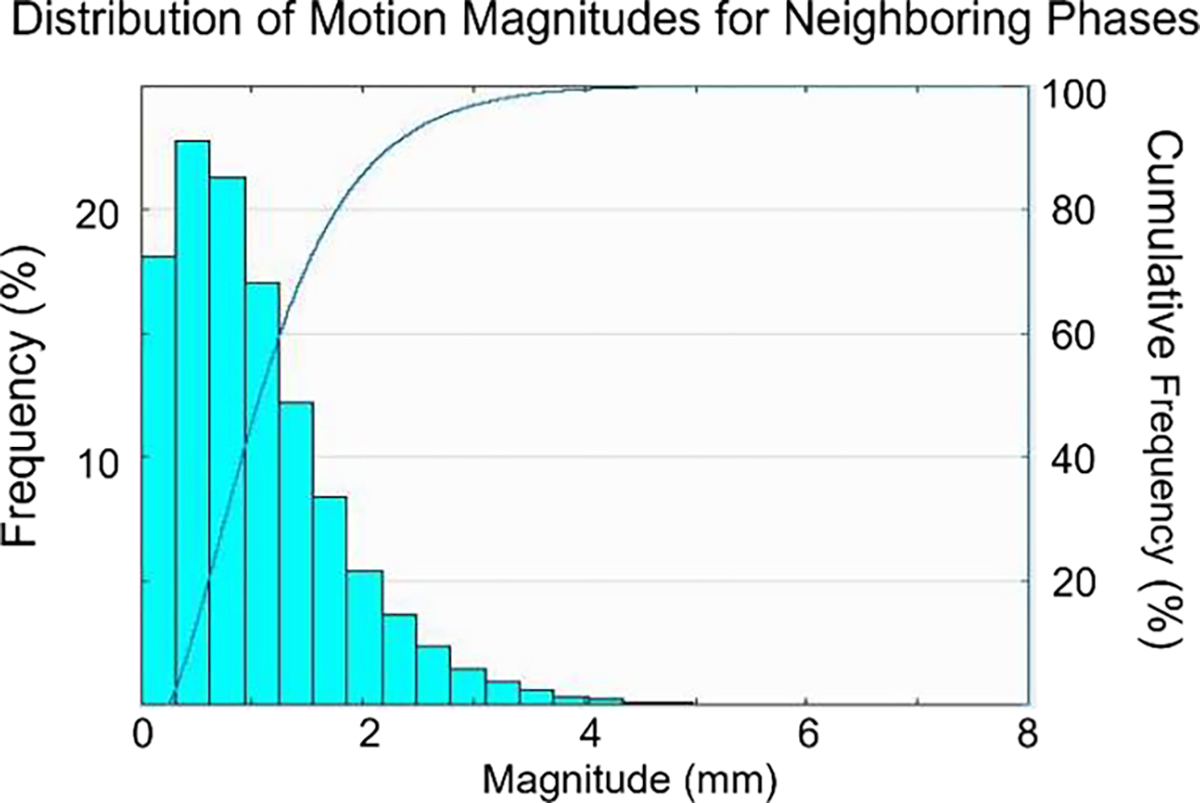
Motion magnitudes for voxels on the stomach surface between neighboring gastric motion phases. The x-axis represents the magnitude of motion (unit: mm); the y-axis on the left is the observed motion frequency, and the y-axis on the right indicates the cumulative probability of motion.

Figure 4 shows the differential and cumulative frequencies for gastric motion magnitudes of stomach surface points between neighboring contractile phases. The majority (97.1%) of the voxels had total motion magnitudes of less than 3 mm, with 87.9% of motions less than 2 mm. Only 0.2% of the voxels moved more than 5 mm. Overall, these measurements indicate that the motions between successive phases are small. This motion is significantly slower than that due to breathing, with 87.9% of the points moving less than 1.1 mm/sec and 97.1% of voxels moving less than 2.2 mm/sec due to contractile forces.

These preliminary observations were used to form the basis for the prediction model and parameters tested. Based on these observations, we can state that a subset of two phases past the current estimated one at any point in time should capture the motion of the stomach to an accuracy of better than 1 mm for at least 91.4% of all points.

### 3.2 Prediction Model Evaluation

**Figure 5.**
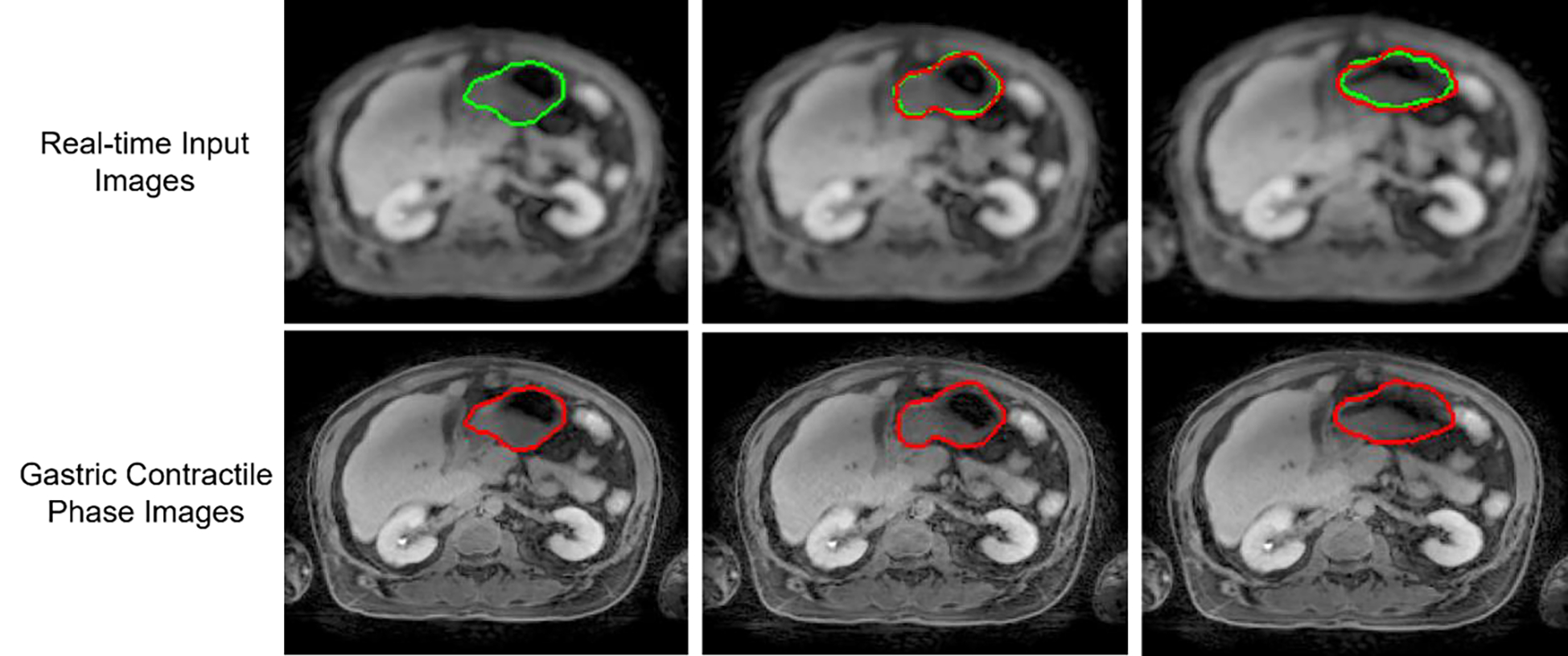
1^st^ row: input images with stomach contours from predicted phases (green) overlaid with the ground truth contour (red); 2^nd^ row: the ground truth phase image for the input image with stomach contours (red). The 1^st^ column showing a correct prediction, and 2^nd^ and 3^rd^ columns showing examples of erroneous predictions.

Figure 5 shows three examples of predicted motion phase and related the ground truth phases, showing a correct prediction and two erroneous ones, respectively. HD95 errors were 0, 2.0 and 2.9 mm for these presented examples.

**Figure 6.**
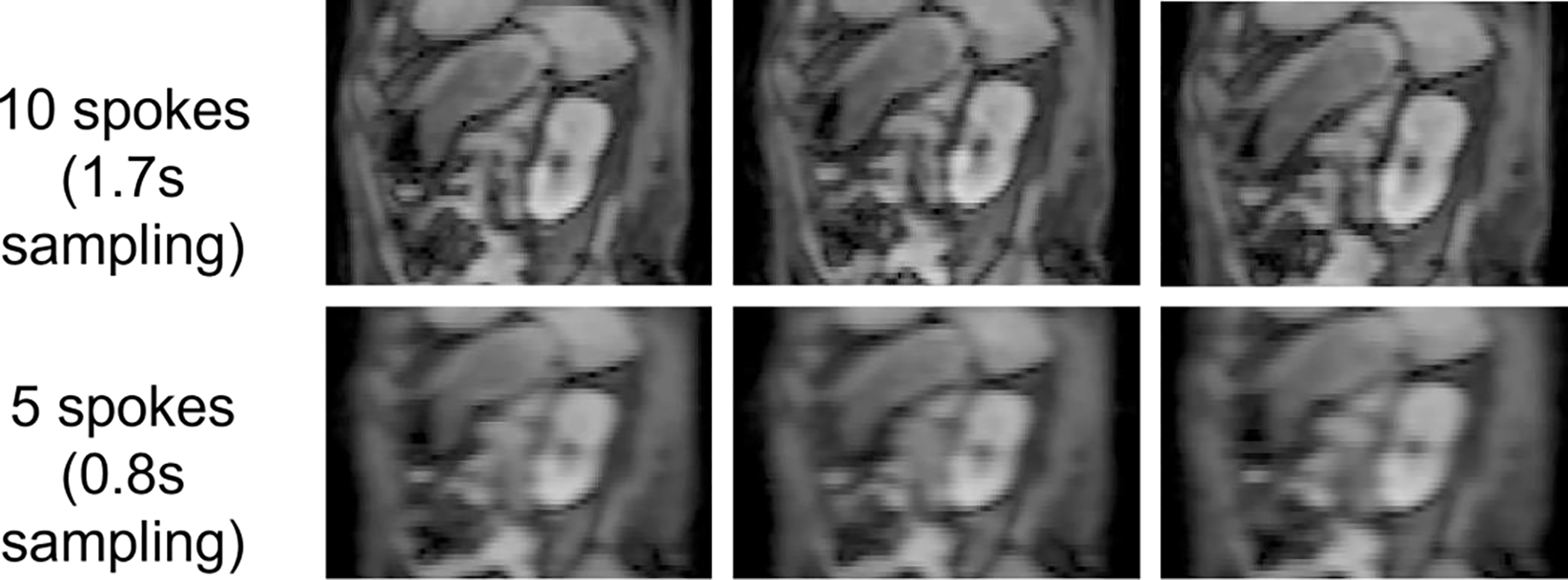
Examples of sagittal planes through input image volumes reconstructed using (top row) 10 and (bottom row) 5 spokes.

Figure 6 shows examples of input images reconstructed using $N_{\text{in}}$ values of 10 and 5 spokes respectively from one example scan, exhibiting noticeable degradation of image quality by reducing the spoke number.

Table 2 lists phase estimation/prediction accuracy and geometric errors with different values of $N_{\text{in}}$. For a 2-minute training window and a $T_{\text{pred}}$ of 5.1 seconds, input images reconstructed using 5 spokes yielded 32% lower prediction accuracy and 0.6mm larger alignment error than input images reconstructed using 10 spokes.

**Figure 7.**
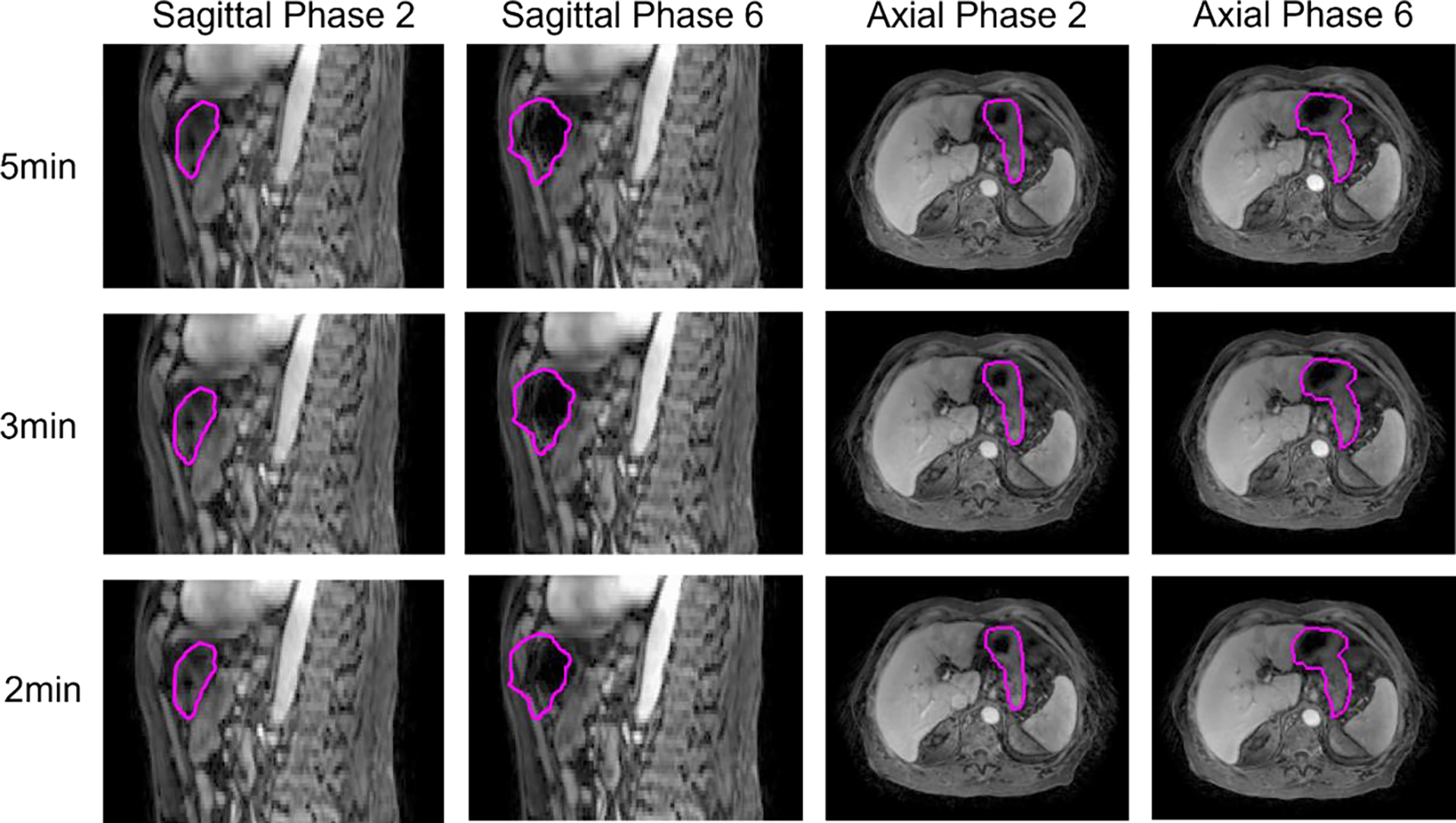
Example reconstructed gastric motion phase images from 5, 3 and 2 minutes of training data from an example patient. The left two columns are sagittal planes for two different motion phases, and the right two columns are corresponding axial planes. The 1^st^, 2^nd^ and 3^rd^ rows represent $T_{\text{train}}$ values of 5, 3 and 2 minutes, respectively. The magenta line is the stomach contour.

Figure 7 shows a random selected example of reconstructed gastric motion phase images using $T_{\text{train}}$ periods of 5, 3 and 2 minutes respectively. Limited degradation in image quality is observed for phase images generated with reduced training times. Compared to phase images of 5min, the main differences observed are streaking artefacts at the edge of the body. The mean SSIM values comparing these 4 slices between 2-to-5-minute and 3-to-5-minute training times were 0.98 and 0.99, respectively.

Prediction accuracy and geometric error for these three different training time windows (with 10 spokes of real-time input image reconstruction) with a $T_{\text{pred}}$ of 5.1 s are listed in Table 3. We can see that the differences in alignment accuracy as well as prediction accuracy using different training time windows are less than 1%. Furthermore, reducing the training time window from 5 to 2 minutes had minimal impact on resulting geometric accuracy. These results supported the use of 2 minutes for training at the start of imaging sessions, a time that is manageable for sampling to generate images for patient positioning and/or treatment adaptation decisions on MR-guided linear accelerators.

**Figure 8.**
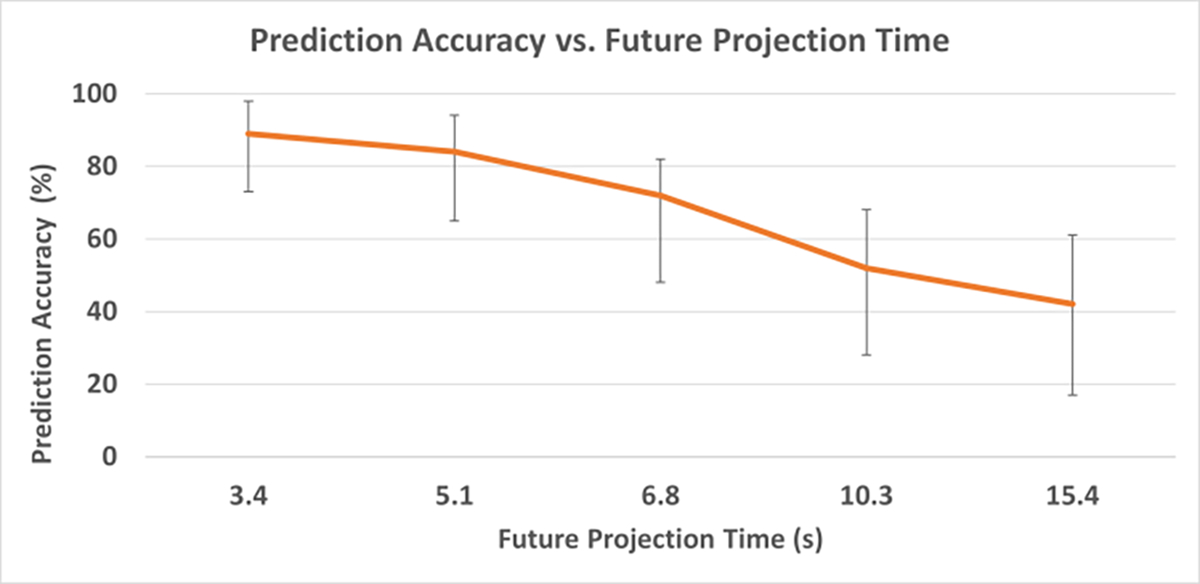
Prediction accuracy for 5 different $T_{\text{pred}}$. The error bars show the range of values across the 25 examinations

**Figure 9.**
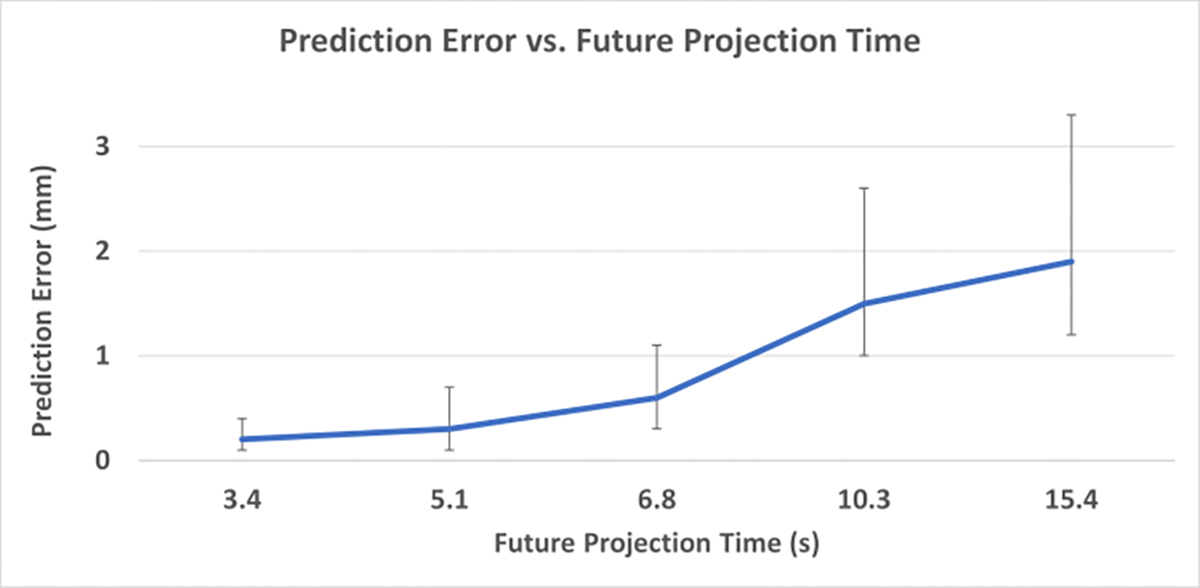
Geometric accuracy (HD95) for 5 different $T_{\text{pred}}$. The error bars show the range of values across the 25 examinations

Phase prediction accuracy results with a $T_{\text{train}}$ of 2 minutes and $N_{\text{in}}$ of 10 spokes for different $T_{\text{pred}}$ are plotted in Figure 8. The prediction accuracy (Figure 9) was 84±6% at $T_{\text{pred}}$ of 5.1 seconds, and 72±8% at 6.8 seconds. For $T_{\text{pred}}$ of 5.1 s, the model yielded an average prediction error of 0.3±0.1 mm; the average error was 0.6±0.2 mm at $T_{\text{pred}}$ of 6.8 s, while the largest prediction error from all examinations was still under 1.1 mm. The prediction error increased to 1.9±0.4 mm (0.9 – 3.8 mm) with $T_{\text{pred}}$ of 15.4 seconds.

In short, with a 2 min training window and 10 spokes for input image reconstruction, the model can achieve submillimeter accuracy in gastric contraction monitoring, which is significantly lower than the average contraction motion magnitude of 3.6±0.5 mm (2.8 – 4.7 mm) quantified by HD95 and likely acceptable for most clinical implementation strategies. Of note, while our average phase prediction accuracies were on the order of 80% across our optimized parameters, the residual phase prediction uncertainties did not translate into significant geometric inaccuracies due to the slow bulk motion between successive phases.

The average computation time for breathing motion identification and correction is approximately 2.5 s on a computation platform with 16 Intel Xeon Gold 6134 CPUs @3.20GHz without any GPU acceleration. The time for the model to estimate and predict gastric contraction motion is less than 1s. The total latency time of the model is estimated to be less than 3.5 s, indicating the feasibility of supporting dynamic contractile motion monitoring with 5 seconds forward projection to support any gating or dynamic treatment adaptation strategies.

## 4. Discussion

A gastric motion prediction model was developed, allowing real-time prediction of stomach contraction. The model takes advantage of the observed short-term regularity of gastric motion in our patient population. Our analysis demonstrates that the model could achieve an average prediction error of 0.3mm with future projection time of 5.1 seconds showing the potential of the model for gastric motion management during treatment delivery.

Recent studies have reported significant inter- and intra-fractional motions for gastrointestinal structures. Alba Magallon-Baro et al. have found deformations from stomach contraction and expansion ranging from 5 – 13 mm in the anterior-posterior direction through Principal Component Analysis[12]. Alam Sadegh et al. conducted an initial investigation on both inter- and intrafraction gastrointestinal organ motions during MR-guided ablative radiation therapy of pancreas patients[13]. Even though they studied T2w MRIs for patients with pneumatic compression belts, median intrafraction deformations of 5.5 mm, 8.2 mm and 7.2 mm were still observed for the stomach/duodenum, small bowel and large bowel, respectively. They further demonstrated that accumulated doses for three patients exceeded institutional constraints for stomach and duodenum. These findings demonstrate the importance of gastric motion management during dose delivery.

We have recently published a separate investigation that proposed a treatment monitoring methodology that tracks motions due to breathing as well as, on a separate time scale, slow changes in abdominal configuration [22]. The methodology presented herein follows similar lines, allowing fast tracking of breathing motion in combination with slower monitoring of gastric contraction. Recently, Akdag and colleagues explored a means to manage cardiac (by gating) and respiratory (by tracking) motions during treatment on a MR Linac by similarly taking into account the presumed reproducibility and low rank nature of cardiac motion and the orthogonality as well as different temporal profiles of breathing and heartbeat [25].

This model is founded in large part on the observed short-term regularity of stomach contractions. In our patients, a major likely cause for inter-cycle reproducibility lies in our guidelines that patients refrain from eating food for at least 2 hours before each visit for simulation or treatment delivery.

Our methodology as currently applied does require a step for gastric contractile phase image reconstruction, which can be achieved by pre-treatment scan used for positioning and/or adaptation decisions. The real-time input images can be acquired with a MR-guided Linear accelerator. This methodology needs a model of breathing-induced deformations to be established in order to apply corrections to new k-space samples as they are acquired. While it is presumed that this can be accomplished during the time needed to adjust for patient position and/or adapt plans, the speed of this process is dependent on computation infrastructure available. Given the relative sparsity of breathing-induced deformations (redundant spatial and temporal information), as well as the availability of prior representations of patient-specific breathing (e.g., from simulation), future methodologies may be implemented to more efficiently model breathing-related deformations quickly at the start of a treatment fraction by limiting the degrees of freedom necessary to scale models to newly measured breathing states.

One important obstacle for a motion prediction model is having enough future prediction time to overcome the latency for including data acquisition and processing as well as the subsequent gating of the treatment beam or movement of the multileaf collimator for tracking. Reported latencies for machine interactions for gated treatments range from 0.2 to 0.5 seconds[14]. The image reconstruction, breathing motion correction and computation time to predict motion states using our model currently occur within 3.5 seconds which, according to our measured results, should be sufficiently fast to support motion management with sub-mm accuracy for gated or dynamically adjusted treatments.

While our investigation generated full volumetric reconstructions for each monitored breathing state, our experiments demonstrated that a single pair of coronal and axial images, properly positioned to maximally intersect the stomach, is sufficient for accurate determination of contractile phase. As such, in theory our k-space derived model could be adapted to orthogonal cine MRI sampling when coupled with appropriate (and existing) breathing management.

Among the 25 examinations we studied, one scan was found that exhibited a noticeable change of configuration due to gas passing through the stomach. The supplementary material presents 3 videos showing reconstructed gastric motion phase images using 3 different time windows of this scan, clearly illustrating how the gas is changing the stomach configuration. Despite the transient configuration changes due to gas, the temporal frequency pattern of stomach contraction remained constant across the scan for this subject. The change of the stomach configuration would significantly affect gastric motion prediction with our current method. Although this situation is rare (1 out of 25 samples), we may conduct future studies to improve the prediction model by updating the gastric motion phase model during the treatment in the background as new spokes are sampled. Other future investigations may include evaluation of dose accumulation with and without the incorporation of contractile motion to simulate the impact of motion on dose to tumors and normal tissues in the proximity of the stomach.

## 5. Conclusion

A gastric motion prediction framework was established by taking advantage of observed short-term regularity of gastric contractions. Our proposed prediction model can achieve sub-mm accuracy in motion prediction with sufficient future prediction time to account for computation as well as nominal latency for interaction with a linear accelerator control system. This prediction framework is easy to implement and is shown to have the potential to support gastric motion monitoring and possibly management during real-time MR-guided radiation therapy.

## Figures and Tables

**Table 1.** Phase numbers and corresponding phase angle centers for 10 reconstructed gastric motion phase images

Phase Number	Phase Angle Center
1	−0.9*π*
2	−0.7*π*
3	−0.5*π*
4	−0.3*π*
5	−0.1*π*
6	0.1*π*
7	0.3*π*
8	0.5*π*
9	0.7*π*
10	0.9*π*

**Table 2.** Phase accuracy and geometric accuracies different $N_{\text{in}}$ values (5 and 10) following 2minutes $T_{\text{train}}$ and 5.1 second $T_{\text{pred}}$ for motion model development.

	Estimation Accuracy (%)	Prediction Accuracy (%)	Estimation Error (mm)	Prediction Error (mm)

**10 Spokes**	90±4 range: 77–96	84±6 range: 68–93	0.2±0.1 range: 0.1–0.5	0.3±0.1 range: 0.1–0.7
**5 Spokes**	68±11 range: 42–81	57±11 range: 37–75	0.6±0.2 range: 0.3–1.4	0.9±0.2 range: 0.5–2.1

**Table 3.** Phase prediction accuracy and alignment error (HD95) using three different $T_{\text{train}}$ values, along with 10 spoke reconstructions for surrogate image volumes.

	Estimation Accuracy (%)	Prediction Accuracy (%)	Estimation Error (mm)	Prediction Error (mm)

**5min**	91±3 range: 83–96	84±5 range: 75–93	0.2±0.1 range: 0.1–0.4	0.3±0.1 range: 0.1–0.6
**3min**	90±4 range: 78–96	84±6 range: 71–93	0.2±0.1 range: 0.1–0.5	0.3±0.1 range: 0.1–0.6
**2min**	90±4 range: 77–96	84±6 range: 68–93	0.2±0.1 range: 0.1–0.5	0.3±0.1 range: 0.1–0.7
